# Effective Spesolimab Treatment for Generalized Pustular Psoriasis Masquerading as Acute Generalized Exanthematous Pustulosis: A Case Report

**DOI:** 10.7759/cureus.81254

**Published:** 2025-03-26

**Authors:** Kazuyoshi Iijima, Yuko Akiyama, Itsumi Mizukawa, Megumi Hirabayashi, Yoshihiro Kuwano

**Affiliations:** 1 Department of Dermatology, Teikyo Mizonokuchi Hospital, Kanagawa, JPN

**Keywords:** acute generalized exanthematous pustulosis (agep), differential diagnosis, generalized pustular psoriasis (gpp), spesolimab, spevigo

## Abstract

Generalized pustular psoriasis (GPP) is a rare and severe variant of psoriasis, often posing diagnostic challenges due to its clinical overlap with acute generalized exanthematous pustulosis (AGEP), a severe drug-induced hypersensitivity reaction. Spesolimab, a monoclonal antibody targeting the interleukin 36 (IL-36) receptor, provides rapid symptom relief for GPP flares, but real-world data remain limited. We report a case of GPP complicated by overlapping features with AGEP, highlighting the importance of precise diagnosis and the efficacy of spesolimab.

## Introduction

Generalized pustular psoriasis (GPP) is a rare but potentially life-threatening inflammatory disorder characterized by the acute onset of painful erythematous plaques covered with sterile pustules [1]. Systemic symptoms such as fever, malaise, and fatigue are frequently observed. Without prompt and appropriate treatment, patients may develop severe complications, including sepsis, disseminated intravascular coagulation, and multi-organ failure [1].

The exact etiology of GPP remains unclear; however, triggers such as certain medications, infections, and the abrupt withdrawal of systemic corticosteroids have been reported [2]. Moreover, mutations in interleukin-36 receptor antagonist (IL36RN), caspase recruitment domain-containing protein 14 (CARD14), and adaptor protein complex 1 subunit sigma 3 (AP1S3) genes have been implicated in the pathogenesis of GPP [3].

Despite the clinical severity and potential for life-threatening complications, no globally standardized treatment guidelines for GPP currently exist [4]. Owing to the rarity of the disease and the unpredictable nature of its flares, high-quality evidence for therapeutic efficacy remains limited. Consequently, most treatment strategies have been extrapolated from plaque psoriasis management. Conventional systemic agents, such as acitretin, cyclosporine, methotrexate, and TNF-α inhibitors, have been employed; however, their delayed onset of action and inconsistent efficacy during acute GPP episodes limit their suitability for urgent intervention [3].

Clinically, GPP shares significant overlap with other pustular disorders, most notably acute generalized exanthematous pustulosis (AGEP), a severe drug-induced hypersensitivity reaction [1,5]. Although both conditions present with diffuse pustular eruptions and systemic symptoms, their pathophysiology and management differ significantly, underscoring the importance of accurate differential diagnosis.

Spesolimab, a monoclonal antibody targeting the interleukin-36 (IL-36) receptor, represents a novel therapeutic approach specifically designed for GPP. Approved by the U.S. Food and Drug Administration (FDA) in 2022 and subsequently in Japan, the European Union, and China, spesolimab has demonstrated rapid and effective relief of symptoms during GPP flares [6].

This report aims to underscore the diagnostic difficulties arising from the clinical resemblance between GPP and AGEP, and to demonstrate the effectiveness of spesolimab in such complex clinical settings. We present a case of GPP that occurred after antibiotic administration, initially raising suspicion of AGEP due to the temporal association and overlapping clinical features. Histopathological findings confirmed the diagnosis, and treatment with spesolimab led to rapid clinical improvement, reinforcing the importance of accurate diagnosis and timely intervention.

## Case presentation

A 56-year-old woman was prescribed a seven-day course of amoxicillin and carbocisteine for the treatment of cough and pharyngitis. Her medical history included hypothyroidism and a notable episode of intermittent right-hand joint pain lasting several weeks one year prior. She had no prior history of significant dermatologic disease, no history of drug allergies, and no relevant family history. Three days after completing the antibiotic regimen, she developed widespread edematous erythema involving the face, neck, back, abdomen, chest, and extremities. There were no pustular or psoriatic lesions on the scalp or nails, and no mucosal involvement was observed in the oral cavity, eyelids, or genital area. 

**Figure 1:**
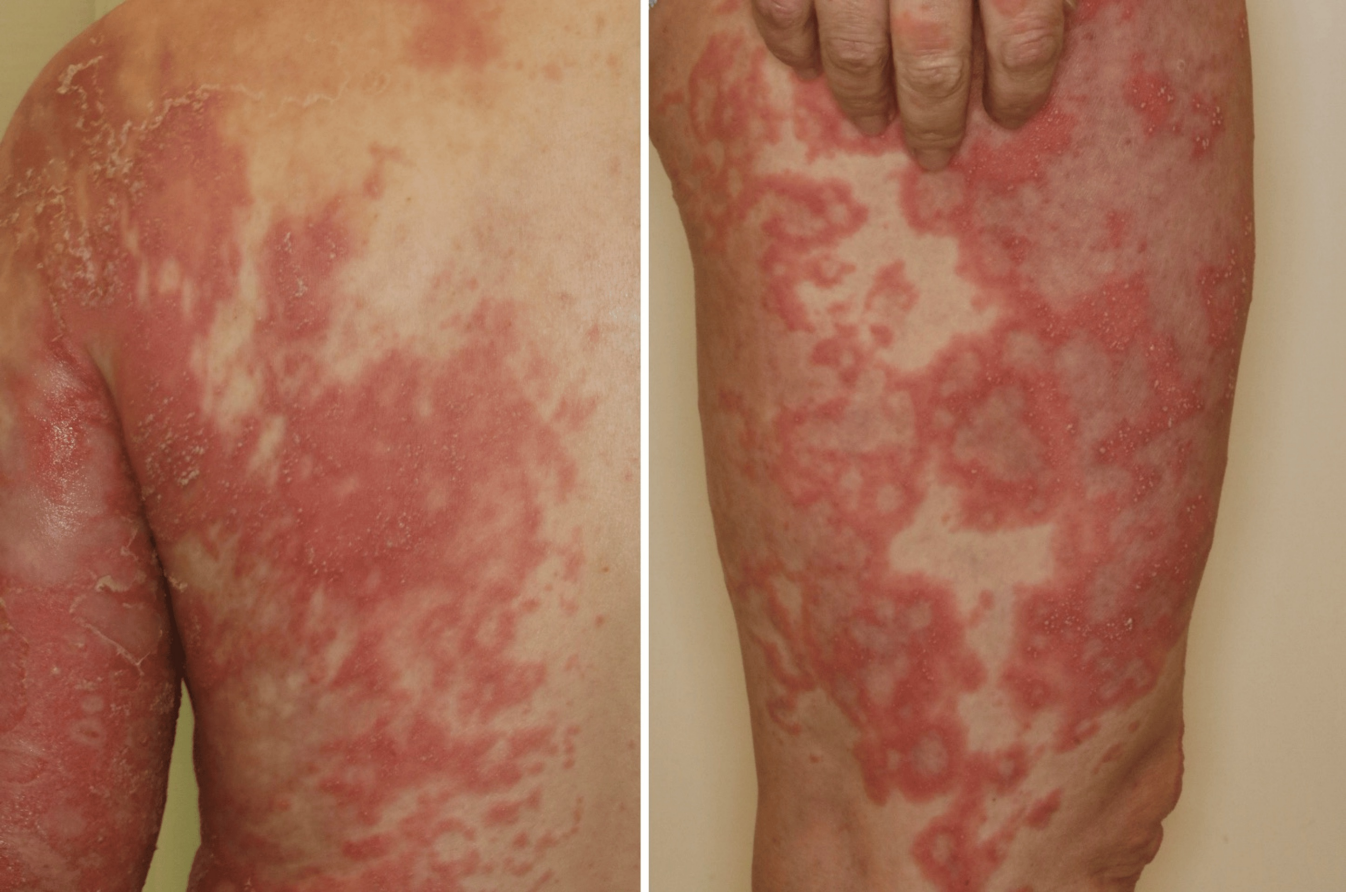
Initial presentation showing widespread sterile pustules and erythematous plaques affecting the face, trunk, and extremities. The pustules exhibited annular and serpiginous configurations, forming lakes of pus.

Despite treatment with oral prednisolone (15 mg/day) and topical corticosteroids, the eruptions persisted and progressively evolved into edematous, annular, bright red plaques, some coalescing into larger erythematous patches. Additionally, pustules measuring 3-5 mm in diameter emerged, fusing into annular and serpiginous patterns to form lakes of pus (Figure 1). 

Due to the worsening of her condition, the patient was referred to our hospital for further evaluation.

On admission, she presented with fever (38°C) and generalized malaise. Laboratory investigations revealed leukocytosis (19,000/μL) and elevated C-reactive protein (CRP) levels (5.11 mg/L). The laboratory data on admission are summarized in Table 1.

**Table 1:** Laboratory findings on admission. WBC: white blood cell; RBC: red blood cell; BUN: blood urea nitrogen; AST: aspartate aminotransferase; ALT: alanine aminotransferase. Normal reference ranges are derived from the American College of Physicians (ACP) guidelines [7].

Parameter	Patient's Value on Admission	Reference Range, Female
WBC (/µL)	19,000	1,560-6,130
RBC (×10⁴/µL)	409	420-590
Hemoglobin (g/dL)	12.4	12-16
Hematocrit (%)	37.2	36-47
Platelet count (×10⁴/µL)	28.6	15-35
Sodium (mEq/L)	139	136-145
Potassium (mEq/L)	3.7	3.5-5.0
Calcium (mg/dL)	8.9	9.0-10.5
BUN (mg/dL)	9.2	8-20
Creatinine (mg/dL)	0.66	0.7-1.3
AST (units/L)	22	0-35
ALT (units/L)	19	0-35
CRP (mg/dL)	5.11	0.0-0.8

**Figure 2:**
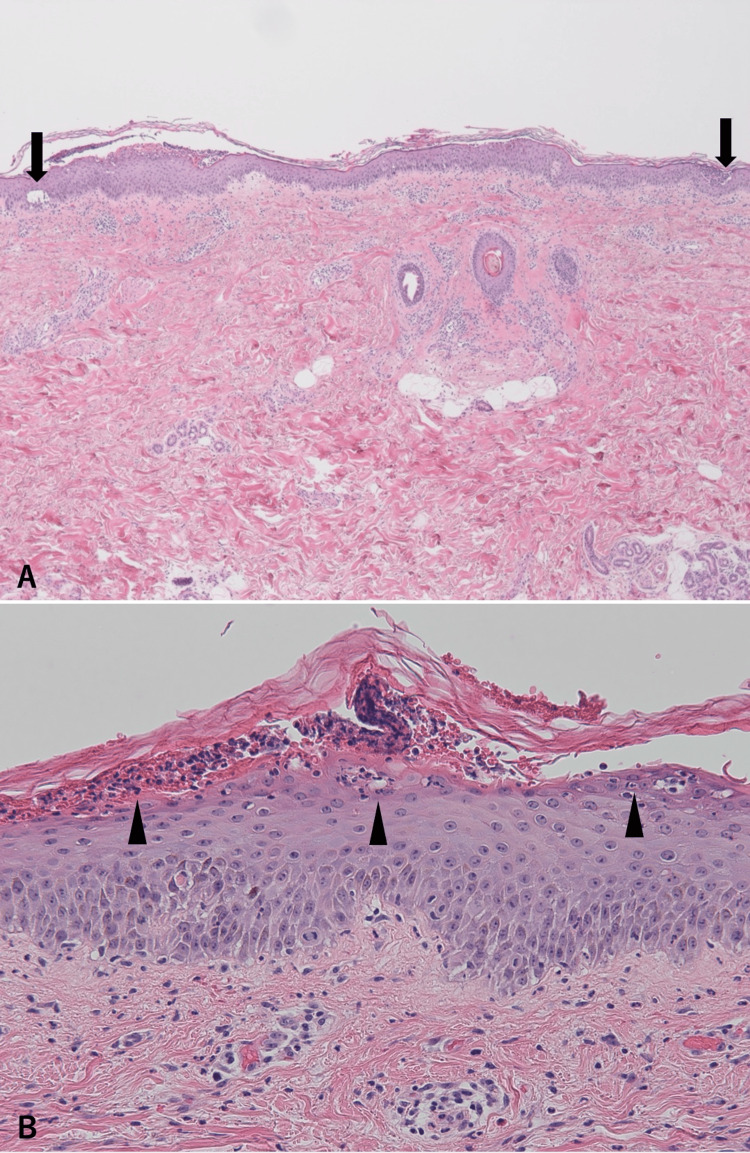
Histopathological findings of skin biopsies from pustular lesions. (A) A biopsy from the forearm reveals subcorneal microabscesses (arrows) without elongation of the rete ridges. (B) Higher magnification shows mild neutrophilic infiltration (arrowheads) throughout the epidermis, and mild inflammatory infiltrates in the dermis, including occasional eosinophils. These findings can be seen in both GPP and AGEP. GPP: generalized pustular psoriasis; AGEP: acute generalized exanthematous pustulosis.

Given the clinical suspicion of both AGEP and GPP, a skin biopsy was performed from an affected area, which revealed subcorneal microabscesses with mild neutrophilic infiltration throughout the epidermis (Figure 2A). Mild inflammatory infiltrates were observed in the dermis, including occasional eosinophils (Figure 2B). There was no evidence of rete ridge elongation.

At this time, the differential diagnosis included both GPP and AGEP. AGEP was initially considered due to the temporal association with recent antibiotic use. However, the clinical course, including the distribution and morphology of the pustules, associated systemic symptoms, and duration, was consistent with both conditions. Histopathological findings from the initial biopsy, including subcorneal pustules and a mild neutrophilic and eosinophilic infiltrate, did not clearly favor either diagnosis. Therefore, a second biopsy was performed from a different lesion to obtain more definitive diagnostic information.

**Figure 3:**
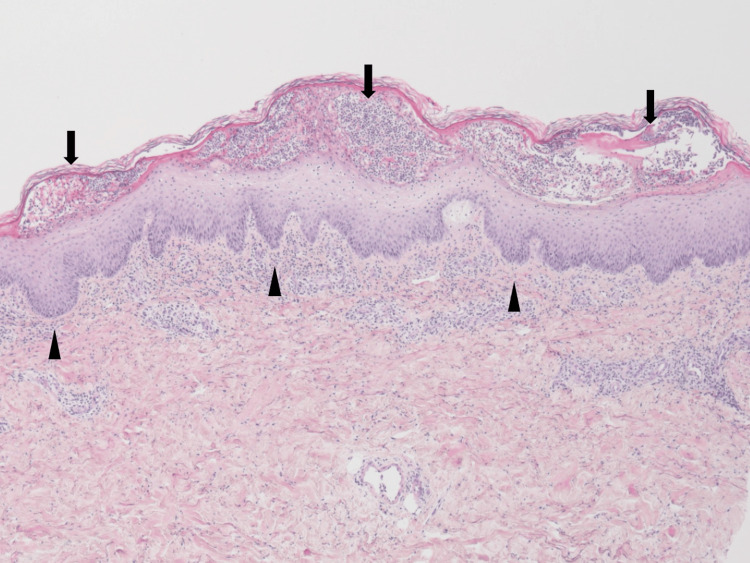
A biopsy from the thigh reveals multiple subcorneal pustules predominantly composed of neutrophils (arrows) and irregular elongation of the rete ridges (arrowheads), which are characteristic histopathological features of GPP and help differentiate it from AGEP. GPP: generalized pustular psoriasis; AGEP: acute generalized exanthematous pustulosis.

A second skin biopsy taken from a separate lesion on the thigh revealed multiple subcorneal pustules predominantly composed of neutrophils, along with irregular elongation of the rete ridges (Figure 3).

Given the persistence of eruptions for 30 days after discontinuation of the suspected drug and the histological findings, a definitive diagnosis of GPP was made. This was the patient’s first episode of GPP. Retrospective analysis of the initial presentation yielded a GPP Physician Global Assessment (GPPGA) score of 3, indicating moderate disease severity.

**Figure 4:**
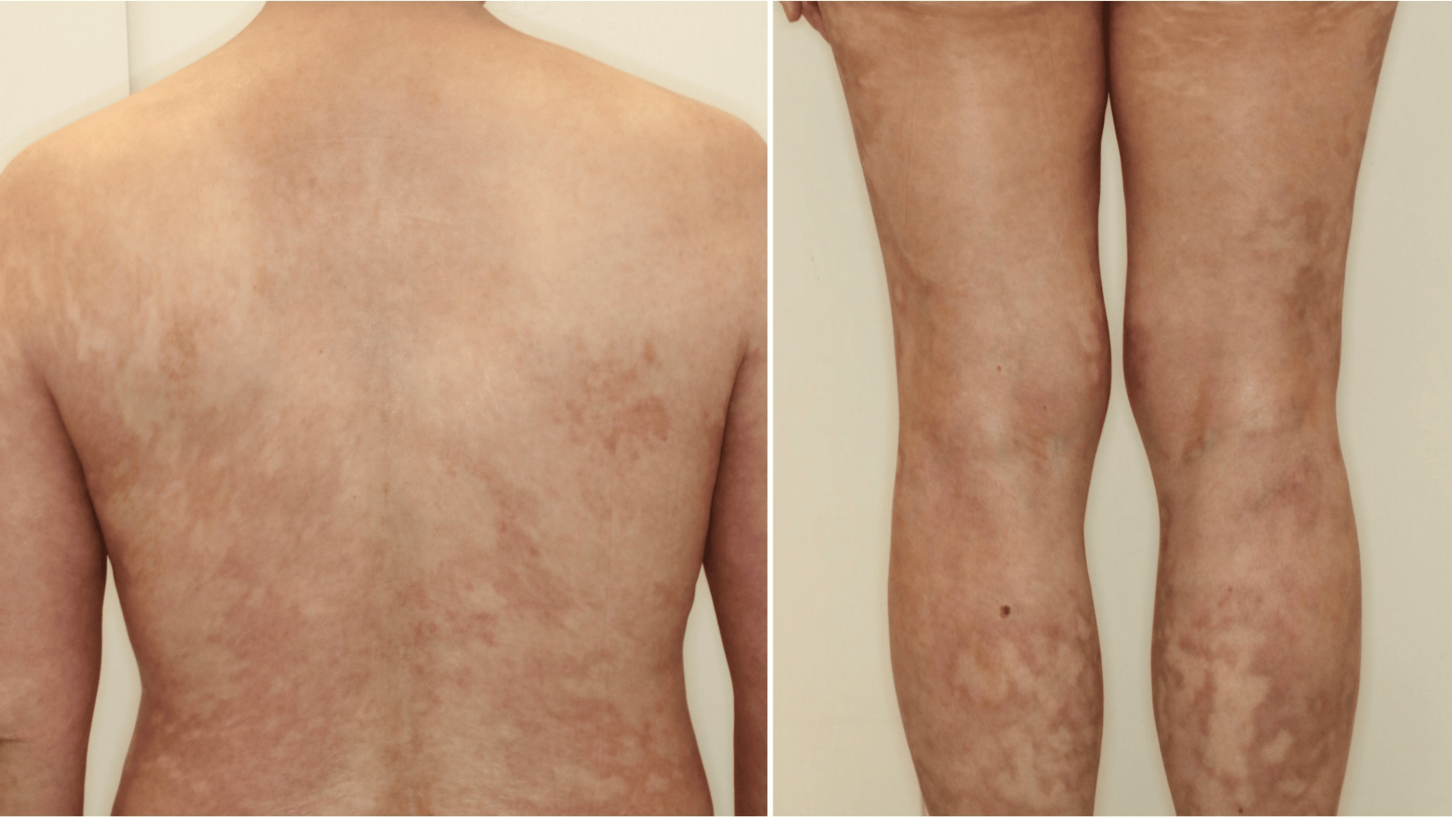
Marked clinical improvement observed one week after the second intravenous infusion of spesolimab. The pustules have completely resolved, and the erythematous plaques have significantly faded, corresponding to a GPPGA score of 0. GPPGA: GPP Physician Global Assessment.

After informed consent was obtained and infectious causes were excluded, the patient was treated with a single 900 mg/body dose of spesolimab, administered two weeks after her initial presentation. As no significant clinical improvement was observed after the first dose, a second dose was administered one week later. Following the second dose, the patient demonstrated marked improvement in her skin lesions, with her GPPGA score improving to 0 within one week (Figure 4). 

She was discharged on a tapering regimen of prednisolone, and no further exacerbation of her condition occurred. To date, the patient has remained free of pustular eruptions for over five months since the resolution of skin lesions.

## Discussion

GPP is a potentially life-threatening inflammatory skin disorder that poses significant diagnostic and therapeutic challenges. Differentiating GPP from AGEP is particularly difficult in patients experiencing their first episode of generalized pustulosis without a history of psoriasis. In the present case, the recent use of amoxicillin and carbocisteine, both known triggers of AGEP, complicated the differential diagnosis. Although AGEP typically manifests within 1-2 days of drug exposure, delayed onset has been reported with certain medications, ranging from 10 to 22 days [1]. While this delayed onset was atypical, the presence of causative drugs, along with the patient’s clinical presentation, initially suggested AGEP.

To aid in differentiation, Yamanaka-Takaichi et al. proposed a scoring system for GPP and AGEP based on six criteria: arthralgia, history of psoriasis or presence of psoriasiform plaques/patches, recurrence or history of pustular disease, history of arthritis, presence of lower-extremity purpura, and the presence of causative drugs [8]. These parameters are assigned scores of 2, 2, 1, 1, -2, and -2, respectively. A total score of -1 or lower suggests AGEP, while a score of 0 or higher supports a diagnosis of GPP. The scoring system has demonstrated high sensitivity (0.85), specificity (1.0), and an area under the curve (AUC) of 0.93 [8]. In our case, the patient’s score of -1, based on a history of joint pain and the presence of causative drugs, reinforced the initial suspicion of AGEP. However, despite discontinuation of the suspected drugs, the patient exhibited a prolonged disease course with persistent pustular eruptions, which was inconsistent with AGEP’s characteristic rapid resolution, typically occurring within approximately 15 days [1]. These discrepancies in clinical presentation and disease progression ultimately supported the diagnosis of GPP.

There are currently no globally standardized treatment guidelines for GPP, and most therapeutic strategies have been extrapolated from plaque psoriasis management [4]. Systemic agents such as methotrexate, cyclosporine, and acitretin have long been used; however, their delayed onset of action and inconsistent efficacy make them suboptimal for managing acute GPP flares [4]. While systemic corticosteroids are generally avoided in pustular psoriasis due to their potential to exacerbate flares, recent evidence indicates that, in well-selected cases with appropriate indications, such as the need for rapid anti-inflammatory effects or bridging to other therapies, they may be used safely with a minimal risk of flare induction [9]. In this case, oral prednisolone at 15 mg/day had already been initiated prior to referral. The dose was tapered gradually, as abrupt discontinuation of systemic corticosteroids is a well-documented precipitating factor for GPP flares [2,10].

Spesolimab, a monoclonal antibody targeting the IL-36 receptor, became the first targeted therapy specifically approved for GPP in 2022. The Effisayil-1 clinical trial demonstrated its rapid efficacy in alleviating acute GPP symptoms and significantly reducing disease severity [11]. Additionally, Sugiura et al. reported that most GPP cases in patients without a history of plaque psoriasis are associated with IL36RN deficiency [12]. While genetic testing was not conducted in this case, previous studies have shown that spesolimab is effective in treating GPP regardless of IL36RN mutation status [11].

Emerging evidence also suggests a potential overlap in the pathogenesis of GPP and AGEP. While IL36RN mutations are primarily associated with GPP, they have also been identified in AGEP. Furthermore, case reports have documented the efficacy of spesolimab in treating AGEP [13,14]. These findings suggest that spesolimab may be a viable therapeutic option in cases where diagnostic uncertainty exists between GPP and AGEP. Future research should focus on elucidating shared mechanisms between GPP and AGEP, refining diagnostic criteria, and evaluating the long-term safety and efficacy of spesolimab to improve patient outcomes.

## Conclusions

Diagnosing GPP in patients without a prior history of plaque psoriasis remains clinically challenging, particularly in distinguishing it from other pustular dermatoses such as AGEP. In this case, histopathological evaluation played a pivotal role in confirming the diagnosis of GPP, allowing for initiation of spesolimab therapy, which led to marked clinical improvement. Given that spesolimab has demonstrated efficacy regardless of IL36RN mutation status, it represents a promising treatment option for managing acute GPP flares. This case underscores the therapeutic potential of spesolimab within the current limitations of available treatment strategies, including its utility in complex diagnostic scenarios. Further research should focus on optimizing diagnostic algorithms, evaluating long-term safety and efficacy, and exploring the broader applicability of spesolimab and other IL-36 pathway inhibitors in pustular skin disorders.
